# Protein local 3D structure prediction by Super Granule Support Vector Machines (Super GSVM)

**DOI:** 10.1186/1471-2105-10-S11-S15

**Published:** 2009-10-08

**Authors:** Bernard Chen, Matthew Johnson

**Affiliations:** 1 Department of Computer Science, University of Central Arkansas, 201 Donaghey Avenue. Conway, AR 72035, USA

## Abstract

**Background:**

Understanding the relationship between the protein sequence and the 3D structure is a major research area in bioinformatics. The prediction of complete protein tertiary structure based only on sequence information is still an impractical work. This paper aims at revealing the hidden knowledge of the sequence motifs and the local tertiary structure.

**Results:**

In this paper, we propose a Super Granule Support Vector Machine (Super GSVM) model to obtain the high quality protein sequence motifs and to predict local tertiary structure information based on purely sequence information.

**Conclusion:**

The proposed model overcomes the innate shortcoming of using the SVM on such a large data set, which is the inherent computational complexity involved in training support vectors for huge datasets including half million of samples. The satisfactory prediction results show the Super GSVM model generates decent protein sequence clusters and has the ability to capture the hidden sequence-to-structure information. This model also has a strong potential in the application of SVMs on other research areas with huge datasets.

## Background

Understanding the relationship between protein sequence and 3D structure is one of the most important research tasks in both biology and bioinformatics researches. Based on many biochemical experiments, it is believed that the sequence is the sole determinate in a polypeptide's structural conformation. This means all the information that is necessary to specify protein interaction sites is embedded into the polypeptide's amino acid sequence [1].

In order to discover the protein sequence-to-structure relationship, Han and Baker used the K-means clustering algorithm to produce high quality protein clusters from protein sequence frequency profiles [2,3]. Subsequently, they used the sequence clusters [4] combined with Hidden Markov Model (HMM) [5] to predict local protein structures. In their work, the clustering algorithm plays the central role in relating protein sequences to local structures. However, the conventional clustering algorithms assume that the distance between data points can be calculated with exact precision. While the distance function is not well characterized, this approach may not reveal the sequence-to-structure relationship efficiently [6].

Support Vector Machines (SVM) [7] has proven their value in various research domains. SVM apply the soft margin idea to allow mislabelled examples for maximization the margin; therefore, SVM has the ability to handle the non-linear classification by implicitly mapping input samples into a higher dimension for maximum-margin hyperplane generation. Under this point of view, SVM may be more efficient to discover the non-linear sequence-to-structure relationship than the K-means clustering algorithm [6]. Nevertheless, due to the high computational cost of SVM, it is not favourable for large datasets [8]. It is almost impossible to model a SVM over half a million data segments, which are then used to generate protein sequence recurring patterns. As a result, SVMs combined with granular computing might be a key step to uncover the secret behind the sequence-to-structure relationship. By using the divide-and-conquer principle, granular computing is able to divide a complex data-mining problem into a series of smaller and computationally simpler problems [9].

In this paper, we explain how to merge the power of SVM and granule computing to uncover the hidden information between the relationship of sequence and structure. A detailed report on local protein structure prediction results based on sequence information is also provided.

## Results

### Super Granule Support Vector Machines (Super GSVM)

**Figure 1:**
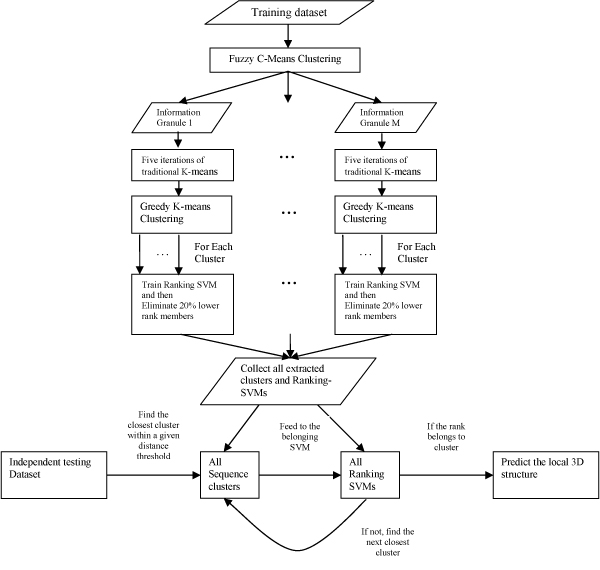
**The sketch of the Super Granule Support Vector Machine (Super GSVM)**.

To perform the true merit of the granular computing combined with the power of the SVM, we propose a new computational model, the Super Granule Support Vector Machines (Super GSVM), in this paper. In the Super GSVM, the large dataset is first softly separated into several information granules by the Fuzzy C-means clustering and then succeeded by the Greedy K-means clustering for sequence cluster generation on each information granule. After that, one Ranking-SVM is built for each sequence cluster to learn the non-linear sequence-to-structure relationship in each cluster. Each Ranking-SVM serves two major purposes: 1. extracting the sequence cluster to generate higher quality protein recurring pattern information; 2. predicting the protein local 3D structure. Figure 1 is the sketch of Super GSVM.

### Protein local 3D structure prediction

In our previous work [11,12], by using the FGK model (described in Method section), we obtained 343 out of 799 high quality sequence clusters using a window size of nine from more than half million sequence segments generated from protein sequence profiles. In this work, we further extract these 799 sequence clusters and improve the high quality cluster number from 343 to 543 by using the Super GSVM model (the improvement results is shown in Table 1). Then we divide these 543 sequence clusters into three groups (excellent, good and fair) based on their secondary structural similarity and use these information with a trained Ranking-SVM model to predict protein local 3D structure from the primary structure (sequence) information. Since the different distance thresholds and the different clustering groups generate distinct prediction accuracy, Table 2 shows a detailed report to indicate the relation between these two factors. Unsurprisingly, the excellent clustering groups always have better prediction accuracy. When the distance threshold is set to 550, the highest prediction accuracy is achieved, which is almost 72%, based on the criteria of the average difference on dmRMSD being less than 1.5 Å. With more restrict (smaller) distance threshold, the prediction usually performs better; however, the prediction coverage (number of predicted segments divided by total number of testing segments, which is 490,426) is getting lower. It is also the reason why we do not show any prediction accuracy with the distance threshold less than 550, no meaningful prediction coverage can be provided. Experimental results show that if we use excellent group of clusters, we are able to predict around 4.5% (21818 out of 490426) sequence segments with 70% accuracy when the distance threshold is set to 850. The analysis between distance threshold and prediction coverage is shown in Table 3. Under smaller distance threshold, the difference of prediction coverage between three clustering groups is not distinct. But since Fair cluster group contains larger number of clusters (287) than Good cluster group (156) and Excellent cluster group (100), while the distance threshold increases, the difference of prediction coverage becomes more obvious.

**Table 1:** The improvement of the number of high quality sequence clusters in each information granule

	G0	G1	G2	G3	G4	G5	G6	G7	G8	G9	Total
Total number of clusters	151	76	95	72	70	133	143	5	48	6	799
**Original FGK-250**											
60%~70%	36	24	24	28	32	31	35	2	15	4	231
70%~80%	21	3	12	4	4	24	20	0	0	0	88
>80%	7	0	7	0	0	4	6	0	0	0	24
**Super GSVM**											
60%~70%	44	30	31	30	42	39	40	3	24	4	287
70%~80%	27	17	17	16	16	27	30	1	4	1	156
>80%	26	2	19	2	1	26	23	0	0	1	100

In this work, we use Super GSVM to generate and extract sequence clusters. We divided the whole training dataset into 10 information granules, the second row shows the number of clusters in each information granule (detail of this information can be found in [11]). The table also show the number of clusters belong to excellent (>80% 2^nd ^structural similarity), good (70%~80% 2^nd ^structural similarity), and fair (60%~70% 2^nd ^structural similarity) clusters before and after the Super GSVM extraction.

**Table 2:** Prediction accuracy on three clustering groups. Since different distance threshold and different clustering groups generate distinct prediction accuracy, Table 2 shows a detailed report.

Distance Threshold	Excellent	Good	Fair
550	71.98%	58.42%	52.89%
600	69.07%	57.16%	52.33%
650	69.08%	57.77%	51.49%
700	69.47%	57.08%	50.31%
750	69.40%	56.82%	49.98%
800	69.85%	56.30%	49.65%
850	70.02%	55.34%	49.37%
900	69.75%	54.78%	48.95%
950	69.53%	53.83%	48.51%
1000	68.88%	53.06%	48.15%
1050	68.26%	52.34%	47.78%
1100	67.63%	51.56%	47.46%
1150	67.09%	51.65%	47.14%
1200	66.54%	50.75%	46.92%
1250	66.11%	50.45%	46.69%
1300	65.73%	50.20%	46.48%

**Table 3:** Prediction coverage on three clustering groups. Table 3 shows the prediction coverage of three clustering groups under different distance threshold.

Distance Threshold	Excellent	Good	Fair
550	0.14%	0.09%	0.20%
600	0.38%	0.27%	0.53%
650	0.78%	0.61%	1.09%
700	1.37%	1.16%	1.95%
750	2.21%	1.99%	3.26%
800	3.26%	3.15%	5.05%
850	4.45%	4.64%	7.46%
900	5.79%	6.51%	10.53%
950	7.19%	8.73%	14.35%
1000	8.61%	11.19%	18.83%
1050	10.05%	13.82%	23.80%
1100	11.46%	16.54%	29.08%
1150	12.77%	19.25%	34.45%
1200	13.95%	21.80%	39.58%
1250	14.99%	24.03%	44.12%
1300	15.83%	25.88%	47.73%

## Discussion

We try to discover the sequence-to-structure relation by predicting protein 3D information, which is mainly focus on dmRMSD, from purely sequence knowledge. Some other tertiary structure knowledge such as torsion angle [1] can also be adapted in our future work as an additional source of 3D structural information. Besides, since the Ranking-SVM also gives ranking information on the target examples instead of simply a yes or no, it is highly possible to develop a strong voting mechanism to generate better prediction accuracy results.

## Conclusion

In this work, we propose a Super GSVM model to discover the hidden protein sequence-to-structure information. We cluster on sequence profiles to find the recurring sequence patterns and evaluate the clusters by secondary structure similarity. We then build a Ranking-SVM for each cluster to improve the secondary structural similarity. Finally, based on the sequence clusters and the corresponding Ranking-SVMs, we predict the tertiary structure of the testing sequences. If the sequence similarity between the testing segment and the existing cluster is verified, we predict the 3D structure of the testing segment should be similar to the representative 3D structure of the sequence cluster. No tertiary structure information is involved in the training process; it is how we carry out the merit of discovering the relation between primary structure and tertiary structure. Although the prediction accuracy is not yet perfect, we open a new door to discovering protein sequence-to-structure information and believe many future works can be applied on our research methods to uncover this mystery. The proposed Super GSVM model is also favourable to many other scientific areas with huge amount of datasets.

## Methods

### Training dataset and independent testing dataset

The training dataset used in this work includes 2710 protein sequences obtained from the Protein Sequence Culling Server (PISCES) [13]. No sequence in this database shared more than a 25% sequence identity. Sliding windows with nine successive residues are generated from each protein sequence. Each window represents one sequence segment of nine continuous positions. More than 560,000 segments are generated by this method and clustered into 800 clusters. The frequency profile from the HSSP [14] is constructed based on the alignment of each protein sequence from the Protein Data Bank (PDB) where all the sequences are considered homologous in the sequence database. Based on the 3D space information obtained from the PDB, we also calculate the distance matrix between all nine *α*-carbons and append this information to each data segment for prediction purposes.

The latest release of PISCES includes 4345 PDB files. Compared with the dataset in our experiment, 2419 PDB files are excluded. Therefore, we regard our 2710 protein files as the training dataset and 2419 protein files as the independent testing dataset, which contains 490,426 segments. Based on 3D space information obtained from PDB, we also calculate the distance matrix between all nine *α*-carbon and append this information to each data segment for testing purpose.

### Primary structure distance and tertiary structure distance (dmRMSD)

For sequence distance, according to [2,15], the city block metric is more suitable for this field of study since it will consider every position of the frequency profile equally. The following formula is used to calculate the distance between two sequence segments [2]:(1)

Where L is the window size and N is 20 which represent 20 different amino acids. F_*k *_(i, j) is the value of the matrix at row i and column j used to represent the sequence segment. F_*c *_(i, j) is the value of the matrix at row i and column j used to represent the centroid of a given sequence cluster.

In order to describe the structure distance, we first introduce Average Distance Matrix (ADM), which records the average for the distance matrices of all the sequence segments in one cluster, using the formula:(2)

Where  is the distance between *α*-carbon atom I and *α*-carbon atom j in the sequence segment k of the length L. Since *α*-carbon indicates the most important location of the protein, we use it to represent the protein center. N is the total number of sequences in the cluster. To calculate the structure distance between the real one and the predicted one, we use dmRMSD [17,18] which is described:(3)

where  is used to represent the predicted sequence cluster's 3D structure and  is the structure information to be predicted. *M *is the number of distances in the distance matrix. Since the window size we use is nine, *M *= 36. In this work, we indicate an successful prediction of local 3D structure if dmRMSD is less than 1.5 Å.

### Secondary structure similarity measure

In the first part of the SUPER GSVM model, we discover protein sequence recurring patters by using a granular clustering approach based on primary sequence structure distance. The quality of the sequence cluster is evaluated by secondary structure similarity. Cluster's average structure is calculated using the following formula:(4)

Where *ws *is the window size and *p*_*i*, *H *_shows the frequency of occurrence of helix among the segments for the cluster in position i. *p*_*i*, *E *_and *p*_*i*, *C *_are defined in a similar way. In order to obtain the representative secondary structure for each position, we use the max function to identify the most frequent appeared secondary structure. For a simplified example: if a cluster with the window size of three contains three members, where their secondary structures are (HEH), (CHH), and (HEH). The representative secondary structure for the first position is H (66%), the representative secondary structure for the second position is E (66%), and the representative secondary structure for the third position is H (100%). Therefore, the average secondary structure similarity is (66%+66%+100%)/3.

Our original idea of using the sequence clusters to predict the tertiary structure is based on the assumption of (1) if the structural homology for a cluster exceeds 70%, the cluster can be considered structurally identical [14], and (2) if the structural homology for the cluster exceeds 60% and is below 70%, the cluster can be considered weakly structurally homologous [15]. We group our sequence clusters into excellent clusters, good clusters, and fair clusters based on the criteria of secondary structure similarity higher than 80%, between 80%~70%, and between 70%~60% respectively. Intuitively, higher quality sequence clusters have better 3D structure prediction power. Therefore, the first step of Super GSVM not only generates sequence clusters, but also extract those clusters into higher quality ones.

### Fuzzy Greedy K-means Model (FGK) Model

Granular computing represents information in the form of aggregates, also called "information granules" [9,16]. For a huge and complicated problem, it uses the divide-and-conquer concept to split the original task into several smaller subtasks to save time and space complexity. Also, in the process of splitting the original task, it comprehends the problem without including meaningless information. As opposed to traditional data-oriented numeric computing, granular computing is knowledgeoriented [9].

**Figure 2:**
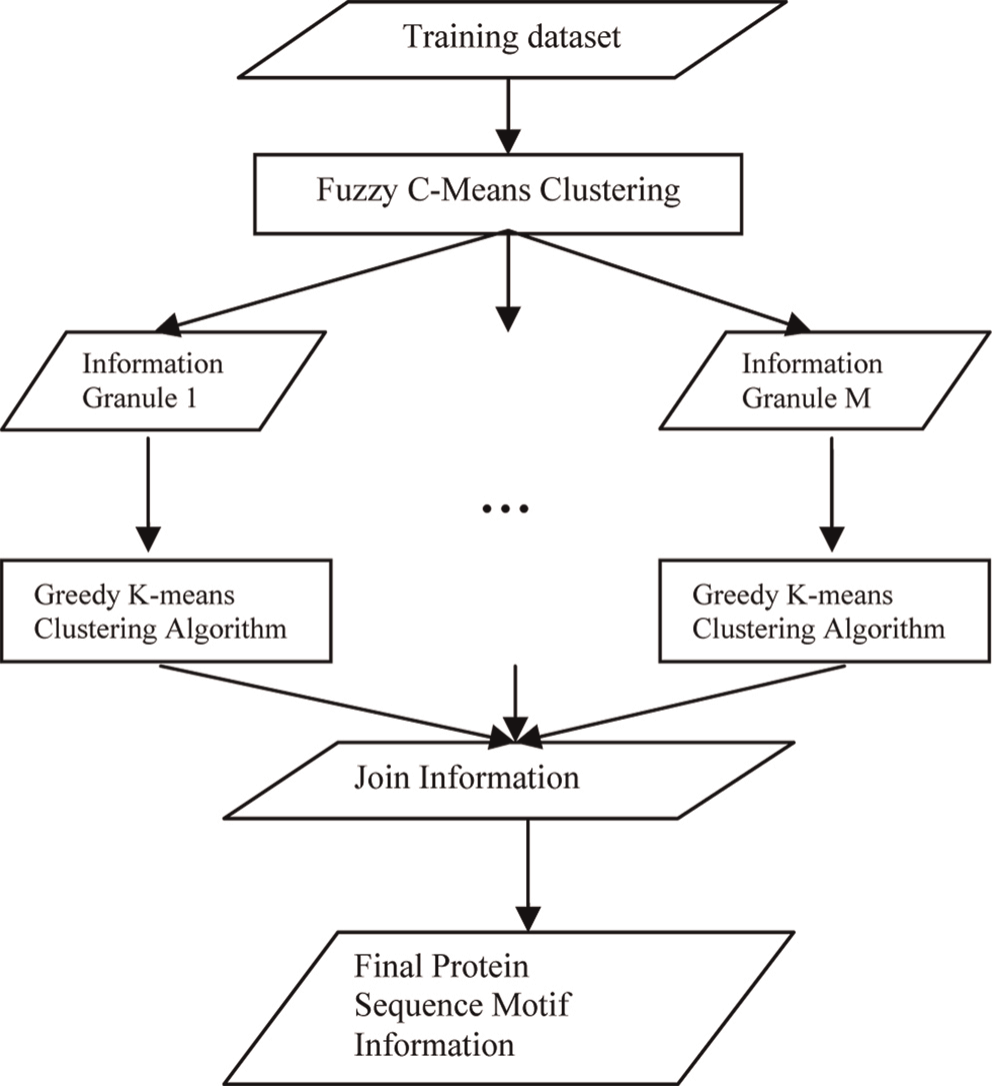
**The sketch of the Fuzzy Greedy K-means (FGK) Model**.

A granular computing based model called "Fuzzy-Greedy-K-means model" (FGK model) is proposed in our previous work [11]. This model works by building a set of information granules by FCM and then applying the greedy K-means clustering algorithm [11], which runs the original K-means clustering five times and then collects the good cluster's centroids as the starting centroids for the sixth round to generate the final information. Major advantages of the FGK model include reduced time- and space complexity, filtered outliers, and higher quality granular information results. Figure 2 shows the sketch of the FGK model. At the first stage, all of the data segments are clustered by Fuzzy C-Means into several "functional granules" by a membership threshold cut. In each functional granule, the new greedy initialization K-means clustering is performed. At the final stage, we join the information generated by all granules and obtain the final sequence motif information.

### Super Granule Support Vector Machine (Super GSVM)

Figure 1 shows the sketch of the proposed Super GSVM model. The whole model can be divided into two parts: 1. Granulating the training dataset and building the Ranking-SVM for each cluster; (Top-down until Collect all extracted clusters and Ranking-SVMs) 2. Local structure prediction produced by the sequence clusters and the corresponding Ranking-SVM. (In the lower part, from left to right)

The first part starts on softly dividing the huge training dataset into several smaller information granules by the Fuzzy C-means clustering algorithm. For each information granule, we perform the Greedy K-means clustering algorithm [11]. Since the cluster size is much smaller than the initial training dataset, we train the Ranking-SVM based on the secondary structure for each cluster and obtain the rank of all members within the cluster. According to our previous report [10], we filtered out 20% of the lower ranking members to generate the clusters with the highest biological and biochemical quality. In this paper, we also filtered out 20% of the lower ranking members to yield higher quality clusters. The improved results are showed in Table 1. Although we cluster the training dataset into 799 clusters, training the Ranking-SVM on all clusters still took us 3 months. Finally, we collect all the sequence clusters and the Ranking-SVM models for the second part: local structure prediction.

The second part of the Super GSVM uses the clusters and the Ranking-SVM generated from the first part to predict the protein local 3D structure from purely sequence information. Please notice that during the first part of the Super GSVM, none of the 3D information is involved. This is mainly because we want to discover the relationship between primary sequence and tertiary structure. For each independent testing sequence segment, we calculate the primary sequence distance (by formula (1)) with all sequence clusters. Within a given distance threshold, if we can find a closest one, we temporarily assign the testing segment to the closest cluster. And then we feed the testing segment into the Ranking-SVM which is generated from the closest cluster to get the rank of the testing segment. If the rank of the testing segment is within the upper 80%, it indicates the segment belongs to this cluster, we then predict that the testing segment should have a similar Average Distance Matrix (ADM) to the cluster. If the rank of the testing segment is not within the upper 80%, it indicates the segment does not belong to this cluster, so we search for next closest cluster and repeat the process. If the testing segment cannot find the closest cluster within the given distance threshold, the testing segment cannot be predicted. Since sequence clusters are considered as the recurring patters or the sequence motifs, the sequence motifs only occur on a handful of locations in the whole protein sequences. That is why we can only emphasize on "Local" structure prediction. Different distance thresholds generate different prediction results. Table 2, 3 and the results section gives the detailed analysis of different parameter set ups.

## Competing interests

The authors declare that they have no competing interests.

## Authors' contributions

BC conceived, designed and performed the study; MJ is a master student in University of Central Arkansas and MJ is directed by BC; BC and MJ wrote computer codes; BC setup the experimental datasets; MJ prepared the protein tertiary structure information; both authors have read and approved the manuscript.
